# Systems biology of meristems: an interview with Teva Vernoux

**DOI:** 10.1186/s12915-018-0591-7

**Published:** 2018-11-01

**Authors:** Teva Vernoux

**Affiliations:** Laboratoire Reproduction et Développement des Plantes, Université de Lyon, CNRS, INRA, ENS de Lyon, UCB Lyon 1, F-69342 Lyon, France

**Keywords:** Plant development, Systems biology, Shoot apical meristem, Plant hormones, Phyllotaxis, Developmental biology

## Abstract

Teva Vernoux is a plant developmental biologist and holds positions as the Director of the Institute for Reproduction and Development of Plants at ENS de Lyon, and as a Research Director at Centre National de la Recherche Scientifique. Teva spoke to us about the need for multidisciplinary approaches to tackle multi-scale problems, how to go beyond a list of genes, and the importance of constructive reviews.

## What are your current research interests?

I’m a plant biologist by training, and in the last 10 years my main focus has been the systems biology of shoot development. The key question is a developmental biology question: how is the coordination of cell identity and behavior achieved? The idea is that signals are sent and received between cells, and how these signals are processed is non-linear, for example, involving positive or negative feedback, which might provide self-organizing properties to the tissues that can explain the dynamics of development.

I use phyllotaxis—the patterning of the shoot apical meristem, which is one of the plant stem cell niches—to tackle this kind of question. There’s been a lot of theoretical work on how the patterning in phyllotaxis could work, and accumulation of experimental data that this is a self-organizing system that can give tissues a capacity for organogenesis. In plants, the signals sent between cells are often plant hormones: my focus has been on a quantitative understanding of the spatiotemporal distributions of such signals in the tissue, and the capacity of cells to read the signals. We try to use classic genomics and genetics on one side—going into the details of which genes are being regulated by which hormones—and, on the other side, a systems biology approach, where you look at the self-organizing behavior of the shoot apical meristem and capture emergent properties of the system. That’s important because it tells you about the properties of the molecular network you’re looking at—just having a list of genes is not going to give you enough information about how they work in a tissue.

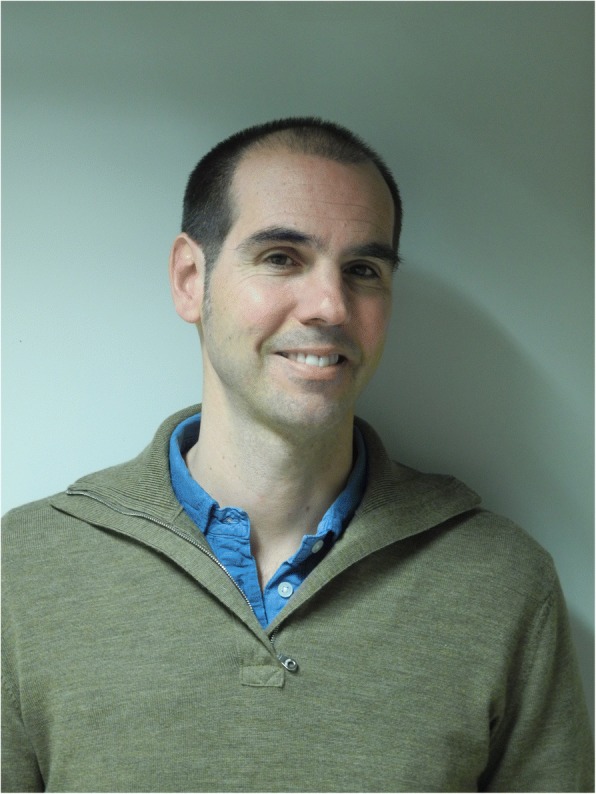



## What are your predictions for the field over the next 5 years?

That’s a tough question! Usually when you have enough of an idea of how things might work that you can make informed predictions, you’re already 5 years into the data, and we’ve only looked at a few of the problems I mentioned. Maybe I can give less of a prediction and more of a wish. Around the 2000s, there was a big emphasis on using systems biology to tackle multi-scale problems—how to integrate data from different scales and to change from one scale to another, which requires a lot of modeling and theoretical analysis. This is pretty difficult and it requires multidisciplinary approaches: my feeling is that these have not developed as much as they should have. My wish would be more of this kind of work, because then you can go after the general principles of development and of systems: the details are only interesting if they’re in a broader context of these kinds of properties.

Part of what is required for this is progress in data acquisition. There are a lot of challenges in data acquisition, particularly when you’re looking across multiple scales, whether molecular networks or the amazing capacity we now have for quantitative dynamic imaging. And then you really need modeling to try and make sense of this huge amount of data: to test ideas and drive the research forward.

One other thing I feel we’re missing somehow at the moment is the capacity to clearly visualize what large data sets are already out there and how we can reuse them. Whilst of course having multiple independently generated datasets can be valuable and add to confidence of findings, there is a risk that time, effort, and money spent be used unnecessarily.

## What motivates you to provide peer review for journals?

I’m now at the stage of my career where, as a Director of an institute, I have more administrative work to do, which takes me a bit away from research. So the first reason for doing peer review is that it helps me to really follow the latest research and to know what is happening in-depth, not just the glimpses that you sometimes get at conferences. It also forces me to go into all the details of the research! That ties in to the second thing that motivates me to review articles—contributing to making the story better and more accessible to a broad range of people. It’s important to focus on the facts in the paper to be able to criticize, but with the definition of a criticism as something that helps make the work better and a constructive filter between the first version and the audience.

## What changes, if any, would you make to the current system of peer review?

One problem that I often see, both as a reviewer and an author, is reviews that are not really fair—they go too far and ask for things that aren’t reasonable. There’s no efficient way in the system yet to turn this kind of review into something more positive for the author. Depending on the journal, editors sometimes follow these kinds of reviews too closely. In my recent experience in reviewing papers notably for *eLife*, I’ve found the “cross-review” system—where there is discussion between reviewers to try to extract what’s most important—is a very interesting contribution to how we do peer-review. It to some extent allows for balancing reviews and ensures that the view that emerges is more of a community vision, rather than from a single person, and helps to reduce these kinds of unnecessarily aggressive reviews.

But these sorts of challenges are complicated by the fact that there are more and more journals and papers! We receive invitations to review papers from an amazing number of sources. I review quite a lot of papers, but I have to turn a lot of invitations down because there’s simply not the time to be able to go into sufficient depth and do a good job.

## Have you had any memorably good or bad experiences of peer review, as an author or as a reviewer?

As a reviewer I would say I often have good experiences—I can’t really think of bad ones. To me a good experience as a reviewer is when the criticisms are all constructive and all go in the same direction.

As an author I obviously have had good and bad experiences. The best thing is when people really like your work and you get a set of reviews that are all positive. One notable bad experience I still remember is from quite early on in my career. I was working on glutathione in cell cycle control and we had a paper showing that there was an effect of glutathione concentration on cell cycle activity. I remember one of the reviewers saying, “OK, you’ve shown that glutathione is important, but water is important as well.” Anybody can make that comment on any type of research—it’s not constructive and it doesn’t help to make the paper any better. I can laugh about it now but it was difficult at the time! We couldn’t really respond to it satisfactorily because it was just a judgment call and there was little to actually argue against. This is one example where the editor didn’t really consider that this was going beyond what a reviewer should be saying.

**Twitter:** @teva_vernoux

**Website:** http://www.ens-lyon.fr/RDP/spip.php?rubrique20&lang=en.

